# Expression of an Endo-β-1,4-glucanase Gene from *Orpinomyces* PC-2 in *Pichia pastoris*

**DOI:** 10.3390/ijms12053366

**Published:** 2011-05-24

**Authors:** Xin Jin, Nan Meng, Li-ming Xia

**Affiliations:** Department of Chemical Engineering and Bioengineering, Zhejiang University, Hangzhou 310027, China; E-Mails: guyin018@gmail.com (X.J.); mengnan17@163.com (N.M.)

**Keywords:** endo-β-1,4-glucanase, *Pichia pastoris*, heterologous expression, neutral cellulase, induction medium

## Abstract

The endo-β-1,4-glucanase gene *celE* from the anaerobic fungus *Orpinomyces* PC-2 was placed under the control of an alcohol oxidase promoter (AOX1) in the plasmid pPIC9K, and integrated into the genome of a methylotrophic yeast *P. pastoris* GS115 by electroporation. The strain with highest endo-β-1,4-glucanase activity was selected and designed as *P. pastoris* egE, and cultivated in shaking flasks. The culture supernatant was assayed by SDS-polyacrylamide gel electrophoresis and showed a single band at about 52 kDa. Furthermore, the recombinant *P. pastoris* egE was proved to possess the ability to utilize sodium carboxymethyl cellulose as a carbon source. The recombinant endoglucanase produced by *P. pastoris* showed maximum activity at pH 6.0 and temperature 45 °C, indicating it was a mesophilic neutral endo-β-1,4-glucanase, suitable for denim biofinishing/washing. Further research was carried out in suitable fermentation medium in shaking flasks. The most favorable methanol addition concentration was discussed and given as 1.0%. After methanol induction for 96 h, the endo-β-1,4-glucanase activity reached 72.5 IU mL^−1^. This is the first report on expression and characterization of endo-β-1,4-glucanase from *Orpinomyces* in *P. pastoris*. The endo-β-1,4-glucanase secreted by recombinant *P. pastoris* represents an attractive potential for both academic research and textile industry application.

## 1. Introduction

Endo-β-1,4-glucanase (EC 3.2.1.4, EG) hydrolyses the cellulose chains internally, providing new chain ends for β-glucosidase in a synergistic manner [1]. Consequently, there has been a rapid growth in demand for endo-β-1,4-glucanase, especially those that possess particular biochemical properties, e.g., neutral characteristics [2,3] or high specific activities [1,4–6]. For effective bio-stoning of denim fibers, complete hydrolysis of the cellulose polymers (processive cellulase action) is unnecessary, and a limited number of random endoglucanase-mediated cleavages within the polymer are sufficient [7]. Most EG enzyme producers, such as *Trichoderma reesei*, always secrete acidic EGs, their pH profile was 4.0–5.5 in general [8–13]. While neutral EGs are superior for applications in denim washing/finishing in the textile industry, due to its superior denim finishing properties, such as low backstaining, as well as its mild reaction conditions [3,13]. Therefore, it is of great interest to obtain high-yield strains with neutral EG activity for its huge potential in practical application.

Anaerobic fungi, first described in 1975 by Orpin [14], are present in the gastrointestinal tract. They produce cellulolytic enzymes with a specific activity much higher than that found in aerobic fungi [14–17]. This remarkable high specific activity draws great attention. However, anaerobic fungi will most likely not be used in industrial enzyme manufacturing because of their slow and strictly anaerobic growth requirement. So far, several genes’ coding for cellulases or hemicellulases, have been cloned and sequenced from anaerobic fungi [18–24]. The endo-β-1,4-glucanase gene *celE* from *Orpinomyces* PC-2 was among them. We have previously cloned the 1.3 kb fragment and expressed in *E. coli* BL21 (DE3) using pET28a(+) as vector. It was a GH5 (glycoside hydrolase family 5) endoglucanase for the high amino acid homology with the endoglucanases from this family, such as CelB29 from *Orpinomyces joyonii* (98.0% identities) [15]. We also have characterized the expressed recombinant EG and determined its optimal pH and temperature for its specific activity to be pH 6.0 and 45 °C, respectively, indicating that it may be a neutral endo-β-1,4-glucanase. However, the SDS-PAGE analysis demonstrated that the protein molecular mass was only approximately 44.0 kDa, which is similar to the deduced molecular mass of the CelE lacking the noncatalytic repeated peptide domain (NCRPD). This implied that the heterologous protein was sensitive to proteolytic cleavage, which was also reported in another study for anaerobic microbe cellulases expressed in *E. coli* [25,26]. Moreover, the intracellular expression in *E. coli* was not a good choice in light of economic benefits. It is therefore desirable to over-express the anaerobic fungi cellulase using a eukaryotic expression system. The methylotrophic yeast, *Pichia pastori*s may be a good choice. As a eukaryote, *Pichia pastoris* has many advantages as a higher eukaryotic expression system such as protein processing, protein folding, posttranslational modification, while being as easy to manipulate as *E. coli* or *Saccharomyces cerevisiae*. As yeast, it shares the advantages of molecular and genetic manipulations with *Saccharomyces*, and it has the added advantage of 10- to 100-fold higher heterologous protein expression levels. These features make *P. pastoris* very useful as a protein expression system [27,28]. In most research, *P. pastoris* is a unique expression system for producing high levels of recombinant proteins [28–35].

Anaerobic fungal strain *Orpinomyces* PC-2 produce multiple EGs [4]. To our knowledge, none of these EGs has been expressed in *P. pastoris* in previous research. In this paper, we report the high expression of an endo-β-1,4-glucanase gene *celE* from the anaerobic fungus *Orpinomyces* PC-2 in *P. pastoris* GS115. Some properties of the heterologous expressed enzyme were also investigated and discussed.

## 2. Results and Discussion

### 2.1. Expression of *celE* Gene in Recombinant *P. Pastoris*

Through sequence analysis of the target gene (accession number: U97153) to the *cel29B* from *Orpinomyces joyonii* and *celB* from *Orpinomyces* PC-2, we concluded that the first 20 amino acids were the signal peptides, the catalytic domain was from M21 to P376, and the last 101 amino acids consisted of the linker and NCRPD sequences. Because the *P. pastoris* expression vector pPIC9K contained the α-Factor secretion signal, the PCR primers were designed to exclude the coding region of the signal peptide. The isolated *celE* gene was about 1.3 kb; it was sequenced and found to have a 99.8% similarity with the reported sequence [20]. There were three mutations, one was in the NCRPD, the other two were silent mutations. It was then ligated to pPIC9K plasmid to generate the expression vector pPICE. Double digestion of pPICE with *EcoR*I and *Not*I released a 1.3 kb fragment corresponding to the size of the isolated *celE* gene, while single digestion by *Sal*I released the 10.5 kb fragment, the same size of the expression vector. In addition, nucleotide sequencing revealed that the genes were inserted in frame into the plasmid.

**Figure 1.**
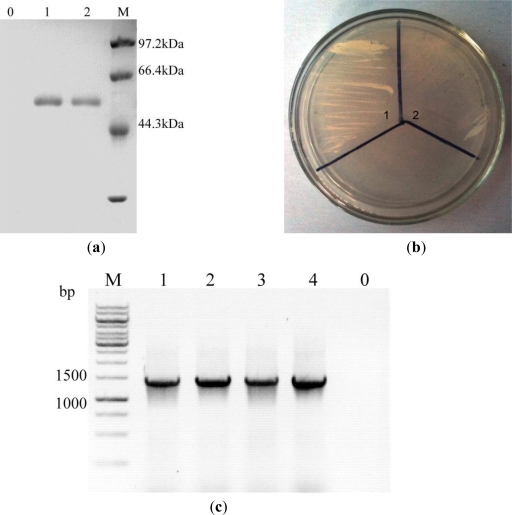
(**a**) SDS-PAGE analysis of the culture supernatant of recombinant *P. pastoris* strain egE. Lane 0: the samples of *P. pastoris* transformed with pPIC9K at 24 h of cultivation; Lanes 1–2: the samples of *P. pastoris* strain egE taken at 24 h, 12 h of cultivation, respectively. Lane M: the protein molecular marker; (**b**) CMC utilization by recombinant *P. pastoris*. The *P. pastoris* strain harboring pPICE (1) or empty plasmid pPIC9K (2) was inoculated in YSM medium to examine the CMC utilization. The CMC-containing plate was incubated at 30 °C for 60 h; (**c**) PCR verification of the *celE* gene integrated in the chromosome of *P. pastoris* egE. Lane 1–4: 1, 4, 6, 10 generations of the *P. pastoris* egE; Lane 0: *P. pastoris* transformed with pPIC9K. Lane M: DNA marker.

After *P. pastoris* transformation, thirty *P. pastoris* transformants with the highest resistance to G418 were selected and subjected to genomic DNA isolation. The PCR assay and nucleotides sequencing were performed, which confirmed that all the transfomants contained an integrated *celE* sequence in the genome DNA. Enzyme activity was then further determined, after induction in shaking flasks in BMGY and BMMY medium. With induction by 0.5% methanol for 48 h, all the recombinant strains expressed and secreted endo-β-1,4-glucanase into the culture broth. Among them, three strains exhibited relatively high production of the recombinant protein, and one of the strains, designed as *P. pastoris* egE, was selected for further study. The endo-β-1,4-glucanase activity reached 35.1 IU mL^−1^ within 48 h after 0.5% methanol induction in shaking flasks. The continuous production of CelE by transgenic *P.pasoris* due to the expression of the *celE* gene was also confirmed by SDS-PAGE analysis of culture supernatants of after growth for 12–24 h in BMMY broth. The total protein profile of transgenic *P.pastoris* on SDS-PAGE showed an apparent single band. It was estimated to be 52 kDa from its mobility relative to standard proteins by SDS-PAGE, similar to the predicted molecular mass (51.5 kDa) (Figure 1a).

To examine the effect of carbon source on endo-β-1,4-glucanase production, we grew the highest EG producing strain *P. pastoris* egE on the YSM medium containing sodium carboxymethyl cellulose (CMC) as the carbon source and compared it to the control strain with empty plasmid pPIC9K. Firstly, the two strains were streaked onto YPD plates and grew for 60 h. Single colonies were then inoculated into 25 mL YPD liquid medium in 250 mL Erlenmeyer flasks, and shaken at 30 °C, 220 rpm. 10 μL of cell culture was streaked on the plates when the OD_600_ was 0.8. The *celE*-expressing transformant grew very quickly, while the control strain hardly grew on the plate (Figure 1b). These results suggest that *P. pastoris* egE expressing and secreting the endo-β-1,4-glucanase CelE acquired the ability to utilize CMC as the carbon source.

Furthermore, to verify the presence of the transformed gene in the chromosome, the *P. pastoris* egE was cultured for multiple generations. The chromosomal DNA was then extracted from different generations of the strains as templates. PCR verification of *celE* gene indicated that the gene has been stably integrated into the chromosome (Figure 1c).

### 2.2. Properties of Expressed Endo-β-1,4-glucanase (EG)

**Figure 2.**
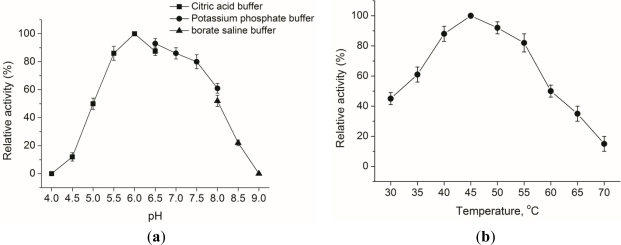
Effects of pH (**a**) and temperature (**b**) on EG activity for *P. pastoris* transformant egE. Each value is means of three replicates. Error bars indicate standard error.

At 40 °C, the effect of pH on EG activity for the hydrolysis of CMC was determined. The result showed that the optimal pH of EG was at pH 6.0 (Figure 2a) and the activity at pH 5.5 and pH 7.5 was more than 80% of that at pH 6.0. At pH 6.0, EG activities were measured at various temperatures to determine the optimal temperature. The result showed that the optimal temperature for the enzyme of interest expressed in *P. pastoris* was 45 °C (Figure 2b). The activity at 40–55 °C was more than 80% of that at 45 °C, but decreased rapidly outside this temperature range. In addition, the pH and temperature optima of the enzyme activity were consistent with those of enzymes expressed from *E. coli*. Depending on these results, the endo-β-1,4-glucanase expressed in *P. pastoris* GS115 can be classified as a mesophilic neutral endoglucanase, which can be used as a good source for industrial applications, especially for denim biofinishing/washing.

As a supplement study, we have expressed both the truncated (lacking the NCRPD) and full-length CelE in *P. pastoris* GS115 and found little difference between the enzymes in terms of activity and optimal pH and temperature. The NCRPD has been found in two groups of proteins with different functions. The NCRPD found in aerobic bacteria/fungi binds cellulose or other carbohydrates, and is usually named CBM (carbohydrate binding motif). In anaerobic fungi, the NCRPD functions as a dockerin domain or docking domian [4,36,37]. It is generally believed that NCRPD is not required for the catalytic activity of the enzyme. Instead, the domains in the anaerobic fungi are always responsible for the assembly of the multiple scaffoldins to be formed as the cellulosomes. This established role of NCRPD is consistant with the finding that the domain has little effect on the catalytic activity or properties of expressed CelE. However, we did find a slight enhanced stability of the enzyme produced from *P. pastoris* when the full-length sequence was used. Therefore the full-length enzyme was subsequently used for all the experiments.

**Figure 3.**
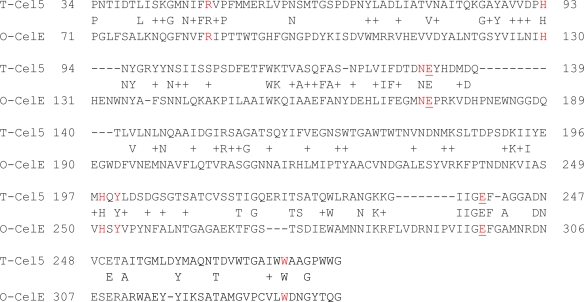
Sequence alignment between *Thermoascus auranticus* endoglucanase (T-Cel5) and *Orpinomyces* PC-2 CelE (O-CelE). The eight strictly conserved residues among GH5 endoglucanases are shown in red. The catalytic proton donor (E133 in T-Cel5) and the nucleophile (E-240 in T-Cel5) are shown in red and underlined. Other identical amino acids between the two proteins are also shown and similar amino acid residues are indicated with the + symbol.

We also compared the expressed CelE with the *Thermoascus aurantiacus* Cel5A, a GH5 member whose crystal structure has been previously determined. A previously reported study has shown that eight different residues are completely conserved among the GH5 enzymes [38]. These include the catalytic proton donor (Glu133 in Cel5A), the neiboring Asn132 and the nucleophile Glu 240. Five other conserved residues are His 93 and Trp273 (implicated in substrate binding), His 198 and Tyr 200 (in interaction with the nucleophile Glu240) and Arg49 (which forms H-bonds with conserved Asn132, His198 and Glu240). As shown in Figure 3, these eight conserved residues are conserved in the CelE protein.

### 2.3. CelE Production by Transgenic *P. Pastoris* in Shaking Flasks

**Figure 4.**
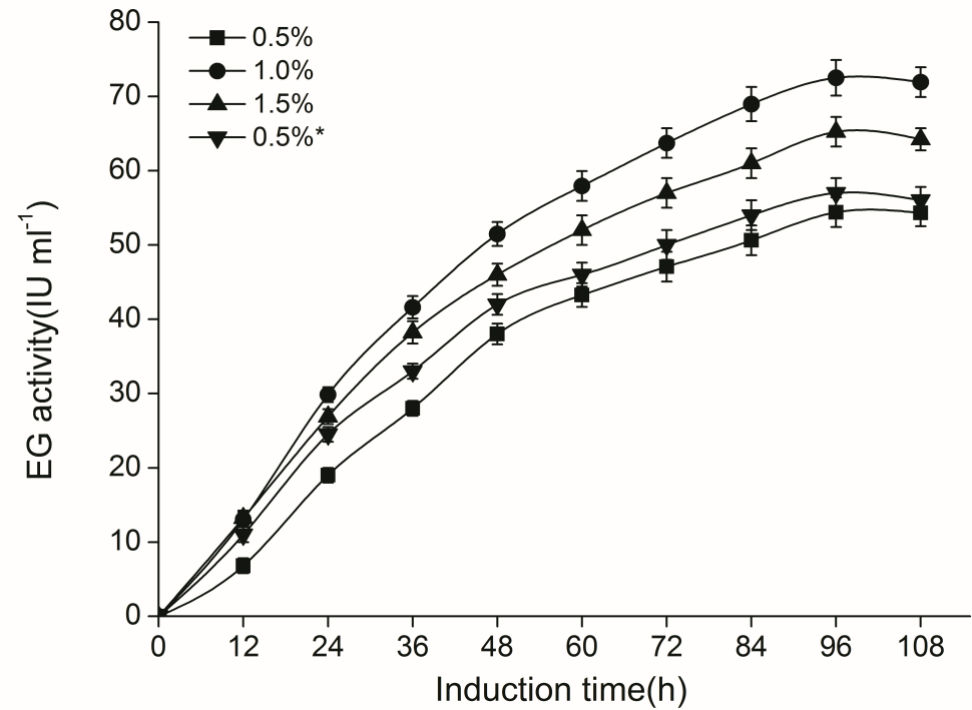
Effect of methanol addition concentration on CelE enzyme production in *P. pastoris* GS115 transformed with pPICE plasmid. The enzyme activity was detected in the liquid BCG medium. Methanol was supplemented every 24 h to a final concentration of 0.5% (squares), 1.0% (circles) or 1.5% (up-triangles). The BMMY medium induced by 0.5% methanol was taken for comparison (down-triangles). Data shown are the means of three independent experiments for each strain.

Recombinant *P. pastoris* strain egE was cultivated in BCG medium in shaking flasks. Meanwhile, different methanol addition concentrations were discussed, and the endo-β-1,4-glucanase activity in supernatants was assayed every 12 h after methanol induction (Figure 4). We noticed that the enzyme production had barely changed when cells were induced by 0.5% methanol in BMMY medium and BCG medium, reaching a level of 57.3 IU mL^−1^ and 54.4 IU mL^−1^ respectively, after 96 h induction, demonstrating that the BCG medium was also appropriate for enzyme production. It should be emphasized here that the BMMY medium is a defined medium in common use, while the BCG medium is a complex medium designed in this study, with corn steep liquor and glucose as the main components. Like BMMY medium, BCG medium contains 100 mM potassium phosphate buffer. The enzyme production was increased significantly more rapidly in BMMY medium than in BCG medium in the first 12–24 h, while the EG accumulation in the broth became closer later. In this sense, the BCG medium could replace the BMMY medium. It is possible that the glucose in BCG medium may prime the cells at the early stage for increased production of the heterologous enzyme at later stages. This was consistent with determination of cell number: about 1.5 times more cells were grown in BCG medium than in BMMY medium at 24 h. Although glucose would repress the synthesis of methanol metabolizing enzymes [39], it had been already exhausted in the first 24–30 h induction time, having little impact on enzyme expression. Besides, the minimal media FBSH has also been tried for induction but was found to have lower enzyme production (39.5 IU mL^−1^) after induction by 0.5% methanol for 96 h.

Methanol accumulation inside the broth has previously been shown to negatively influence the production level [40]. In the study of added methanol concentrations, we found that at 1.0%, the enzyme activity rapidly rose after methanol supplementation and reached its peak (72.5 IU mL^−1^) when inducted for 96 h, which were 33.3% and 11.2% higher than the 0.5% (54.4 IU mL^−1^) and 1.5% (65.2 IU mL^−1^), respectively. The control strain (transformed by plasmid pPIC9K) has no endo-β-1,4-glucanase production at all (data not shown).

According to previous studies, a lower methanol concentration has resulted in a lower cell growth rate and, consequently, a lower yield of recombinant proteins [28,39,41]. However, it is also important to note that the induction of AOX1 promoter may decline at high methanol concentrations due to toxic products of alcohol oxidation having inhibitory effects directly on the AOX enzyme and cell growth in general [42,43]. A substantial higher laccase activity was obtained from recombinant *P. pastoris* at 0.5% than at 1.0% methanol concentration [44].

We have monitored the cells number by OD_600_ every 12 h as well. The OD_600_ for 0.5%, 1.0%, 1.5% methanol concentration were 18.2, 25.7 and 27.1 at 84 h, respectively. The OD_600_ in BMMY medium was 19.8 with 0.5% methanol induction. Thus, low methanol concentration (0.5%) was not adequate for enzyme production. Although a higher concentration (1.5%) was beneficial for cell growth, the EG activity was affected negatively. Induction of the AOX1 promoter by higher methanol concentration could accelerate the cell growth as well as the rate of the protein synthesis, allowing less time for the peptide chains to fold properly [44]. Moreover, the toxic products from rapid metabolism may also be a negative factor.

The GH5 endo-β-1,4-glucanase EGII from *T.reesei* was of good quality in denim finishing and other applications [45,46]. In previous research, Qiao *et al.* [47] has expressed the EGII in *P. pastoris* GS115 using pPIC9K. The EG is a typical acidic cellulase and the optima pH of 5.0. The specific activity of the enzyme was less than 60% than at pH 5. The highest EG activity of recombinant *P. pastoris*/EGII in shaking flasks was only 8.73 IU mL^−1^. Liu *et al.* [48] have also expressed the EGIV from *T.reesei* in *P. pastoris*. The EG activities of cultivation supernatant of *P. pastoris* EGIV was 2.4 IU mL^−1^. Obviously, these EG activities were much lower than those from our study. Besides, the specificity activity of the crude enzyme from the 96 h fermentation broth (1.0% methanol concentration) was also determined to be 24.7 IU mg^−1^. This was superior to other studies, e.g., Thongekkaew *et al.* [49] expressed the EG from Cryptococcus *sp. S-2* in *Pichia pastoris*, and the specific activity of crude enzyme was 4.93 IU mg^−1^. In another study, the specific activity was 7.7 IU mg^−1^ with the EG from *Volvariella volvacea* [50]. The anaerobic fungi produce EGs with high specific activity which would greatly enhance its catalytic ability, indicating the great potential of recombinant CelE enzyme produced by *P. pastoris*. It is of substantial value for industrial application, such as the textile bio-finishing or paper deinking, which are preferable for the function of EGs. In addition, to the best of our knowledge, this is the first report of functional expression of neutral GH5 endo-β-1,4-glucanase gene from anaerobic fungi in *Pichia pastoris*. Further characterization of the enzyme, including substrate specificity and identification of critical amino acid residues, would be of great interest, and we intend to pursue these studies in the future as our long-term research direction.

## 3. Experimental Section

### 3.1. Strains, Plasmids and Culture Medium

Anaerobic fungus, *Orpinomyces* PC-2, was described by Borneman *et al.* (1989). The fungus was grown anaerobically at 39 °C as previously described [51]. *Pichia pastoris* strain GS115 and plasmid pPIC9K were purchased from Invitrogen Corporation (San Diego, CA, USA). The plasmid pPIC9K was used for expression in *P. pastoris*. Expression of inserts was controlled by the methanol-inducible alcohol oxidase gene (AOX1) promoter. Plasmids were introduced into *E. coli* DH5α to prepare enough amount (about 10 μg) of plasmids required for transformation of *P. pastoris*.

The *E. coli* DH5α was cultivated in LB medium. *P. pastoris* GS115 was cultured in YPD medium for propagation. The minimal media FBSH [52], BMGY and BMMY liquid medium were used for *P. pastoris* recombinants growth, heterologous enzyme expression and screening, as described in the manual of methods for the easy-select *P. pastoris* expression system (www.invitrogen.com/content/sfs/manuals/easyselecte_man.pdf). YSM medium was used to evaluate CMC utilization by recombinant yeast. BCG medium was used for enzyme expression in shaking flasks.

### 3.2. Chemicals and Enzymes

CMC was purchased from Sigma Chemical Co., Ltd (St Louis, USA). Enzymes (*Taq* DNA polymerase and T4 DNA ligase) were purchased from Sangon (Shanghai, China). Protein markers and restriction endonucleases were purchased from Takara (Dalian, China).

### 3.3. Recombinant DNA Techniques

The *Orpinomyces* PC-2 was cultured [51] and isolated were done by the methods of Rozman and Komel DNA manipulation [53]. The *celE* gene was amplified from the genomic DNA by PCR according to its sequence reported [51]. *N*-terminal analysis by SignalP program (http://www.cbs.dtu.dk/services/SignalP/) suggested that the first 20 amino acid residues encoded the signal peptide, which was deleted by PCR method. Primer sequences were: E1: 5′-CGCGAATTCATGAGAGAAATATTCCATCCAAAG-3′, E2: 5′-GCGGCCGCATAAATACCACACCAGTTACCAT-3′.

Amplified PCR products were resolved by 1% agarose gel electrophoresis and ligated into the pMD18-T simple vector. The cloned DNA fragments were verified through DNA sequencing. Gel slices containing the expected size band was excised and extracted with Takara Agarose Gel DNA Purification Kit Ver. 2.0 (Dalian, China). The *celE* gene was inserted into the *EcoR*I and *Not*I sites of the vector pPIC9K to generate the pPICE construct.

### 3.4. Transformation and Transformants Screening

*Pichia pastoris* strain GS115 was grown in 500 mL of yeast extract-peptone-dextrose (YPD) medium for 18 h and prepared for transformation. The plasmid pPICE were linearized and used to transform *P. pastoris* by electroporation (0.75 kV, 25 uF, 200 Ω, SCIENTZ-2B Genepulser; Ningbo, China). Transformants screening of multiple inserts was according to the Invitrogen pPIC9K Expression Manual and antibiotic G418 was used in five concentrations: 1.0, 2.0, 3.0, 4.0 and 5.0 mg/mL. To induce expression and screen the high EG producing recombinants, strains with highest resistence to G418 were selected and taken the procedure respectively: Single clone was cultured in 5 mL YPD medium at 30 °C overnight with shaking at 220 rpm. The cells were then incubated in 250 mL shaking flask containing 25 mL BMGY medium at 30 °C for 16–18 h. Harvest the cells by centrifuging at 1500–3000 g for 5 min at room temperature. To induce expression, decant the supernatant and resuspend cell pellet in 25 mL BMMY medium. The following procedures were according with that of Invitrogen Pichia Expression Kit (Version F). The EG activity was assayed and compared at 48 h. Three replicate experiments were performed for every experiment. From the transformants that exhibited the highest enzymatic activities, one was selected for fermentation and characterization.

### 3.5. Sodium Dodecyl Sulphate-Polyacrylamide Gel Electrophoresis (SDS-PAGE)

After centrifugation, the supernatant samples were loaded on 12% SDS-PAGE gels. After electrophoresis, the gels were stained for 40 min in 30% methanol, 10% acetic acid and 0.1% Coomassie brilliant blue and then destained for 12 h in 30% methanol and 10% acetic acid. For the molecular mass determination the protein molecular weight marker from Takara (Dalian, China) was used.

### 3.6. Endo-β-1,4-glucanase Activity Assay

The endo-β-1,4-glucanase activity of the supernatant samples against CMC substrates was estimated by incubating 1.0 mL of 1.0% CMC-Na solution in 50 mM citric acid buffer (pH 6.0) with 0.5 mL of the diluted crude enzyme solution (about 10–400 fold varying with the enzyme activity, also diluted in 50 mM citric acid buffer) at 45 °C for 15 min. The reaction was terminated by addition of 2 mL of 3,5-dinitrosalicyclic acid reagent and brown color was developed after holding all of the assay tubes in boiling water for 5 min. The contents of the tubes were diluted to 10 ml with distilled water and the absorbance was measured at 540 nm [54]. One unit of Endo-β-1,4-glucanase activity was defined as the quantity required to release 1 μmol of glucose per minute (IU mL^−1^).

### 3.7. Optimal pH and Temperature Condition

The endo-β-1,4-glucanase produced from transgenic *P. pastoris* strains were measured in different conditions. The optimum pH of the expressed endoglucanase was determined at 45 °C at every half point between 4.0 and 9.0 and adjusted by 50 mM citrate buffer solution (pH 4.0–6.5), 50mM sodium phosphate buffer (pH 6.5–8.0) and 50 mM borate saline buffer (pH 8.0–9.0). The endo-β-1,4-glucanase activities were measured at 5 °C intervals between 30 and 70 °C to determine the optimum temperature. The temperature was controlled in a water bath shaker.

### 3.8. Enzyme Production in Shaking Flask Culture

*Pichia pastoris* GS115 transformed with the pPICE plasmid which had highest endo-β-1,4-glucanase activity was inoculated on YPD solid medium at 30 °C for 2 days. Single colony was then inoculated into 25 mL YPD liquid medium in 250 mL Erlenmeyer flasks, and shaken at 30 °C, 220 rpm for 24 h. Then the seed culture were harvested and transferred into 25 mL BMMY/FBSH/BCG medium in the 250 mL shake flasks. Three replicated experiments were performed for every experiment. The inoculum ratio was 10% (v/v), and the flasks were shaken at 30 °C, 220 rpm. Methanol was supplemented every 24 h to a final concentration of 0.5%, 1.0% or 1.5% (v/v).

## 4. Conclusions

The neutral endo-β-1,4-glucanases have wide applications in textile and paper industries. However, most neutral cellulase producers are bacteria or anaerobic fungi [20,55–57], both of which have low producing ability. In this work, we successfully cloned and expressed the endo-β-1,4-glucanase gene *celE* from an anaerobic fungus *Orpinomyces* PC-2 in *P. pastoris* GS115. The yeast strain became able to utilize CMC as a sole carbon source, and continuously secreted the neutral endo-β-1,4-glucanase. In addition, a simple fermentation medium was introduced firstly to replace BMMY medium, which substantially reduced the economic cost for production. After 1.0% methanol induction for 96 h, the EG activity reached 72.5 IU mL^−1^. This newly constructed yeast strain could be widely applicable, especially for denim-staining system and paper recycling scheme, due to its neutral characteristics and high hydrolysis activity towards β-1,4-glucan linkages. To our knowledge, this is the first report of functional expression of neutral GH5 endo-β-1,4-glucanase gene from anaerobic fungi in *P. pastoris*.

## Figures and Tables

**Table 1.** Media for *P. pastoris* cultivation.

**Media**	**Composition**
BMGY	1% Glycerol, 1% yeast extract (YE), 2% peptone, 0.34% yeast nitrogen base without amino acids (YNB), 1% (NH_4_)_2_SO_4_ and 4 × 10^−5^% biotin in pH 6.0 100 mM potassium phosphate buffer
BMMY	0.5% Methanol, 1% YE, 2% peptone, 0.34% YNB, 1% (NH_4_)_2_SO_4_ and 4 × 10^−5^% biotin in 100 mM potassium phosphate buffer
FBSH	26.7 mL/L H_3_PO_4_ (85% stock), 0.93 g/L CaSO_4_, 18.2 g/L K_2_SO_4_, 14.9 g/L MgSO_4_·7H_2_O, 4.13 g/L KOH and adjusted to pH 5.0 by NH_4_OH. Add 0.004% histidine and 0.435 mL of PTM1 trace metal solution (6 g/L CuSO_4_·5H_2_O, 0.08 g/L KI, 3 g/L MgSO_4_·H_2_O, 0.2 g/L Na_2_MoO_4_, 0.02 g/L H_3_BO_3_, 0.5 g/L CoCl_2_, 20 g/L ZnCl_2_, 65 g/L FeSO_4_·7H_2_O, 0.2 g/L biotin and 5 mL H_2_SO_4_)
BCG	1% corn steep liquor, 2% glucose in 100 mM potassium phosphate buffer
YPD	1% yeast extract, 2% peptone, 2% glucose, add 2% agar powder when YPD plate prepared
YSM	0.67% YNB, 2% Sodium carboxymethyl cellulose (CMC-Na), 0.5% methanol, 2% agar powder, pH 6.0
